# The Influence of Temporal Disturbances in EKF Calculations on the Achieved Parameters of Flight Control and Stabilization of UAVs

**DOI:** 10.3390/s24123826

**Published:** 2024-06-13

**Authors:** Jędrzej Szczepaniak, Bogusław Szlachetko, Michał Lower

**Affiliations:** 1 Faculty of Electronics, Photonics and Microsystems, Wroclaw University of Science and Technology, 50-370 Wrocław, Poland; boguslaw.szlachetko@pwr.edu.pl; 2 Sky Tronic Sp. z o.o., ul. Zduńska 10, 51-180 Wrocław, Poland; michal.lower@pwr.edu.pl; 3 Faculty of Information and Communication Technology, Wroclaw University of Science and Technology, 50-370 Wrocław, Poland

**Keywords:** unmanned aerial vehicle (UAV), drone, multirotor, extended Kalman filter (EKF), PX4-ECL, real-time operating system (RTOS), flight stability, magnetic disturbance

## Abstract

This article investigates the causes of occasional flight instability observed in Unmanned Aerial Vehicles (UAVs). The issue manifests as unexpected oscillations that can lead to emergency landings. The analysis focuses on delays in the Extended Kalman Filter (EKF) algorithm used to estimate the drone’s attitude, position, and velocity. These delays disrupt the flight stabilization process. The research identifies two potential causes for the delays. First cause is magnetic field distrurbances created by UAV motors and external magnetic fields (e.g., power lines) that can interfere with magnetometer readings, leading to extended EKF calculations. Second cause is EKF fusion step implementation of the PX4-ECL library combining magnetometer data with other sensor measurements, which can become computionally expensive, especially when dealing with inconsistent magnetic field readings. This can significantly increase EKF processing time. The authors propose a solution of moving the magnetic field estimation calculations to a separate, lower-priority thread. This would prevent them from blocking the main EKF loop and causing delays. The implemented monitoring techniques allow for continuous observation of the real-time operating system’s behavior. Since addressing the identified issues, no significant problems have been encountered during flights. However, ongoing monitoring is crucial due to the infrequent and unpredictable nature of the disturbances.

## 1. Introduction

Miniature Unmanned Aerial Vehicles (UAVs), commonly referred to as drones, are becoming increasingly popular due to their wide range of applications in various fields, such as aerial photography, infrastructure inspection, or package delivery. A precise estimate of the attitude, velocities, and position of a drone is crucial to ensure stable flight and mission safety. To achieve this, a variety of measurement sensors are used onboard the UAV. The most commonly used measurement sensors are three-axis gyroscopes and accelerometers. Additionally, a three-axis magnetometer is used to determine the geographical orientation (yaw angle) and a barometer to determine the relative flight altitude. In this way, measurement systems with names such as 6-DOF (accelerometers and gyroscopes), 7-DOF (like 6-DOF plus barometer), 9-DOF (accelerometers, gyroscopes and magnetometers), and 10-DOF (like 9-DOF plus barometer) are constructed, where DOF stands for Degree Of Freedom. Due to the need to reduce the costs of an individual UAV and its small size, the used sensors are characterized by significant noise and other measurement errors, such as bias or incorrect scaling. Some of these errors are minimized through special calibration procedures [1,2]. However, scaling and bias errors are not constant over time, so regardless of calibration, it is necessary to monitor and compensate for these errors during flight.

The simplest and least computationally expensive algorithms for attitude determination are based on complementary filtering [3,4,5,6,7]. Complementary filters determine the attitude by combining data from accelerometers and gyroscopes. A typical complementary filter consists of two filters with different time constants. A low-pass filter with a small time constant is used to filter high-frequency noise, while a filter with a large time constant is used to filter low-frequency noise. This division is based on the observation that during large changes in angles, i.e., at high angular velocities, the gyroscope bias has significantly less influence on the integration of the gyroscope measurements, allowing for the calculation of attitude changes. However, when the UAV is hovering or in a stable flight phase, i.e., when the angular velocities of drone are small, the estimation of roll and pitch angles based on accelerometer measurements is significantly less erroneous. As a result, the fusion of accelerometer measurements involves assigning appropriate weights depending on the rate of change in angles. Therefore, for small rates of change, a higher weight is assigned to accelerometers and a lower weight to gyroscopes, and for large rates of change, it is the opposite. Complementary filtering algorithms have been developed using Euler angles [7,8] and quaternions [4,6,9,10,11,12]. It should be noted that the fusion of accelerometer and gyroscope measurements does not allow the determination of geographical orientation, that is, the Yaw angle. For this reason, algorithms have been developed that additionally fuse measurements from magnetometers. The attitude thus determined can be used to construct a rotation matrix from the drone body frame to the reference frame (most commonly geographic), which theoretically allows for the calculation of the linear position and velocity of the UAV in the reference frame. Unfortunately, the measurement errors of the accelerometers used cause the calculated position and linear velocity to become useless very quickly. Therefore, complementary filters are currently only used on drones manually operated by the operator in a Visual Line of Sight (VLOS) scenario.

More advanced solutions compared to complementary filters are Kalman filters (KFs). These are estimation algorithms based on Bayes theory [13]. Describing the dynamic state changes in the UAV during flight allows for the correlation of physical variables describing the object (e.g., drone position change, its linear velocities, and accelerations) with measurement data from various sensors. As a result, this allows for the inclusion of measurement and process noise during calculations, leading to an increased precision of the obtained results. In the case of linear KFs, statistical convergence of the calculation results with the real state of the UAV is achieved [13,14]. In the problem of estimating attitude angles, position, and UAV’s own velocity, KFs combine data from gyroscopes, accelerometers, and magnetometers, as well as data from GNSS receivers and many other sensors. An extension of the KF idea are Extended Kalman Filters (EKFs), which allow for describing the system’s dynamics using nonlinear equations. Then, a linear approximation of the derivatives of drone state changes is determined in its current state, leading to the linearization of the equation. The subsequent steps of the EKF algorithm are analogous to those of KF, except that linearization is also applied during the measurement fusion stage [15,16,17].

In recent years, KFs have gained significant popularity due to the availability of cheap and fast microcontrollers equipped with Floating Point Units (FPUs), which greatly simplifies the implementation of calculations. The basic quantities contained in the UAV state for KFs and EKFs are quaternions that represent attitude, position, and velocity in the local or global reference frame. In some implementations, attitude error is used instead of quaternions [18,19,20], resulting in smaller matrix sizes and consequently faster calculations. More advanced implementations add gyroscopes and accelerometers bias to the state, modeled as random walking noise, significantly improving the functioning of EKFs [15,21,22,23,24,25]. The list of quantities describing the EKF state may include additional quantities such as accelerometer scale factor [25,26], Earth’s magnetic field, wind velocity [27], and many others. However, this always results in an increase in the size of calculated matrices, and consequently in greater computational complexity. The evolution of the system state over time is calculated by the EKF based on equations describing the system’s dynamics, considering the previous state and control, as well as the model of process noise. Furthermore, additional sensors measure selected physical quantities. In the case of the availability of these additional measurements, the state of the UAV can be corrected by calculating Kalman gains based on the variance of a given measurement and the variance of the state associated with that measurement. The GNSS receiver is commonly used to correct the UAV state by measuring position and velocities in the global reference frame. This is a widely used measurement. In the absence of a GNSS signal, visual [28,29], LIDAR [2,17,30], or other systems are used.

The Kalman Filter is a recursive algorithm consisting of two steps commonly called state prediction and state correction. The second step of the algorithm is optional in the sense that if there are no new measurements, only the prediction is calculated based on the equations describing the system dynamics and possible control. In our case, an estimation algorithm that implements KF is one of many software threads running on the UAV’s onboard microcontroller. These threads together form our own onboard Flight Control (FC) system. The KF output in the form of current attitude, position, and velocities is used by other threads to stabilize the flight and perform the programmed mission. The FC system assumes that at least the attitude stabilization and speed control threads are running as real-time threads. In other words, a time regime is assumed, in this case a constant, cyclic time interval between successive activations of the attitude stabilizer and the speed controller (and/or position controller depending on the flight phase). A delay in the start-up time of any of these threads results in disturbances in stabilization and changes in the flight path. For this reason, the KF thread should also meet the real-time requirements [31] in order to determine the current state values of the UAV at specific required time intervals. The article presents the results of experimental studies performed on a UAV in which the FC uses the PX4-ECL library [27], which implements a 24-state EKF. The time period required by the FC for up-to-date attitude data to be available is 2 ms and position and velocities every 10 ms. As experiments have shown, the PX4-ECL library on the SMT32F7xx microcontroller running at 216 MHz clock shows deterministic behavior, i.e., the state prediction step calculation time is more or less constant and is about 150 μs, while the correction step calculation time is usually less than 900 μs. The correction step is performed relatively rarely, only a few times per second. Unfortunately, there are cases during flight when the correction step calculation time is longer than the required 2 ms, mostly under 10 ms, but there were observed cases that reached 70 ms. What is worse, when this phenomenon occurs, it is not a one-time event. Then, in each subsequent iteration of the EKF computation loop, the extended correction step times are repeated. This obviously leads to visible flight instability and creates situations that endanger flight safety. Since these situations are very rare, they have not been described in the literature so far. This article investigates the hypothetical causes of this phenomenon and suggests ways to fix it.

Our research concerns some specific flight control software, but this does not limit the generality of the considerations because the ECL library is very popular and widely used in many solutions, both at universities and in the industry. It should be noted that the same autopilots are used for small UAVs and large ones weighing up to several dozen kilograms. The main factor in air accidents involving UAVs, apart from physical damage to drone components, is the autopilot software, especially the EKF filter responsible for calculating the current state of the UAV. All delays or calculation errors lead to loss of flight control.

Loss of flight control in UAVs using autopilots with a built-in EKF filter is a relatively rare phenomenon and can only be observed during flight. The result of such an event may be a UAV disaster, which itself may have far-reaching consequences, including a threat to human life or large material losses. These unexplained cases are rare, but cause concerns and tighten regulations related to the use of UAVs in public spaces. Therefore, solutions to the problem of loss of flight control are intensively sought. In work [32], the authors propose a solution consisting of introducing additional distance sensors whose task is to measure the distance to the ground at several points simultaneously. Assuming a flat ground surface, roll and pitch angles can be calculated from these measurements. As a consequence, the EKF filter obtains an additional source of information, which translates into better estimation of the gyroscope bias and more stable filter operation. In turn, in another work [23], the authors look for a solution by detailed examination of the actual properties of measurement sensors, mainly gyroscopes, accelerometers, and magnetometers. In this way, more precise control of measurement noise in the covariance matrix prediction step can be ensured. Increasing the number of various types of measurement sensors installed on UAVs is considered the right direction to improve the quality of calculations in the EKF filter. This is particularly important when there is a temporary or complete loss of one of the main measurement signals, namely the GNSS signal. Then, the EKF filter can only be based on the fusion of data from the UAV’s own sensors. Depending on flight conditions and the number and type of sensors, the flight can continue or must be interrupted due to possible calculation errors of the EKF filter [2,19,33,34,35].

The main aim of this publication is to provide a detailed analysis of the causes of rare cases of loss of flight control. According to our analysis, computational time delays in the main loop are the main cause of this phenomenon. The work also identifies the cause of delays. This research allows for us to discover that these delays appear in special flight conditions, when the magnetometer readings are inconsistent with the current magnetic field calculated by the EKF algorithm implemented in the ECL software library [27].

## 2. Materials and Methods

This section contains the description of the researched problem (Section 2.1). In Section 2.2, the EKF library used to estimate the state of the UAV is presented. The tool used to analyze the integrity of the UAV real-time operating system is presented in Section 2.3. The methodology of the research is presented in Section 2.4.

### 2.1. Problem Statement

**Figure 1:**
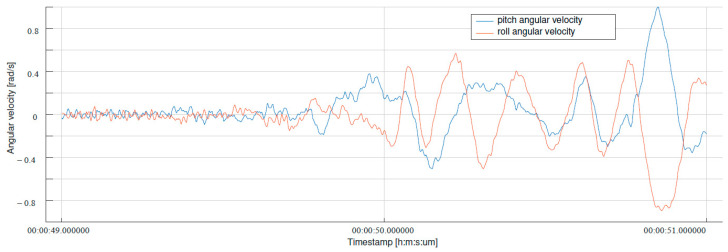
Results of a sample test flight with the disruptions and an emergency landing.

The reliable operation of the UAV stabilization system is a natural expectation of every drone operator. Our tests showed that the UAV we tested occasionally oscillated. The observed phenomenon occurred very rarely and most often lasted a few seconds. The resulting oscillations were generally small and difficult to identify by the operator. In most cases, oscillations could only be observed on archive graphs and were interpreted as a temporary disruption of the flight stabilization process. Although in most situations the disruption was minor and the UAV returned to normal operation, there were cases in which the disruption was large enough to force the operator to make an emergency landing. An example of such a situation is shown in Figure 1.

The graph presented in the Figure 1 shows the angular velocities of the UAV in the roll and pitch axes. As can be seen in the graph, the UAV unexpectedly began to oscillate in both axes. The oscillations were so large that the operator decided to make an immediate emergency landing. The surprising thing was that after the test was repeated, the flight was performed correctly.

The analysis of the observed disturbances did not indicate their source in external factors. A detailed analysis of the flight stabilization process did not reveal any reasons for disruptions in inappropriate responses of the flight stabilization algorithm. Ultimately, it was concluded that the causes of the problem should be sought in the process of generating current attitude values, which are used to stabilize the flight. Particular attention was paid to delays in published attitude values. Due to the fact that the published attitude values are the result of ECL filter calculations, it was decided to analyze the operation of this part of the software.

### 2.2. EKF Model

**Figure 2:**
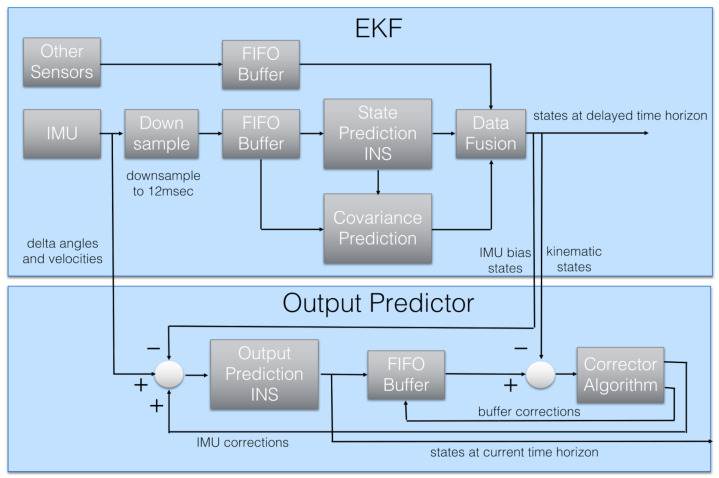
PX4-ECL EKF flow diagram [27].

The Estimation and Control Library [27] is an open-source library that includes the EKF fusion algorithm, which implements the delayed fusion time horizon. This feature allows for the integration of sensor measurement with different delays relative to the Inertial Measurement Unit (IMU). The diagram of a data flow in the EKF algorithm is shown in Figure 2. As one can see, the key data used to predict the state of the EKF come from the inertial sensors: gyroscope and accelerometer. This block is marked—IMU. However, the remaining measurements—marked Other Sensors—are used at the correction stage in the EKF filter—block Data Fusion.

The filter includes a 24-state vector:

- the quaternion defining rotation from the North-East-Down (NED) local earth frame to the Front-Right-Down (FRD) body frame,
- velocity at the IMU in the NED frame,
- position at the IMU in the NED frame,
- IMU gyroscope bias estimates in the FRD body frame,
- IMU accelerometer bias estimates in the FRD body frame,
- Earth Magnetic field components in the NED local frame,
- vehicle body frame magnetic field bias in the FRD body frame,
- wind velocity estimate in the NE frame.

The EKF fusion algorithm is capable of incorporating data from various sensors, including accelerometers, gyroscopes, magnetometers, barometers, global navigation satellite systems (GNSS), downward-facing distance sensors, optical flow sensors, airspeed sensors, and external vision odometry systems.

The state vector of the EKF filter can be written as:

(1) $$x={\left[q,v_{N},p_{N},b_{\omega },b_{a},m_{E},m_{B},w_{NE}\right]}^{T},$$

where

(2) $$\begin{array}{cc} q & ={\left[q_{0},q_{1},q_{2},q_{3}\right]}^{T}, \end{array}$$

(3) $$\begin{array}{cc} v_{N} & ={\left[v_{N},v_{E},v_{D}\right]}^{T}, \end{array}$$

(4) $$\begin{array}{cc} p_{N} & ={\left[p_{N},p_{E},p_{D}\right]}^{T}, \end{array}$$

(5) $$\begin{array}{cc} b_{\omega } & ={\left[b_{\omega x},b_{\omega y},b_{\omega z}\right]}^{T}, \end{array}$$

(6) $$\begin{array}{cc} b_{a} & ={\left[b_{ax},b_{ay},b_{az}\right]}^{T}, \end{array}$$

(7) $$\begin{array}{cc} m_{E} & ={\left[m_{Ex},m_{Ey},m_{Ez}\right]}^{T}, \end{array}$$

(8) $$\begin{array}{cc} m_{B} & ={\left[m_{Bx},m_{By},m_{Bz}\right]}^{T}, \end{array}$$

(9) $$\begin{array}{cc} w_{NE} & ={\left[w_{N},w_{E}\right]}^{T}. \end{array}$$

IMU sensors provide direct measurements of angular velocities $\omega_{x,y,z}$ and linear accelerations $a_{x,y,z}$ within body frames. These measurements are subject to biases and noise. The biases are presumed to remain relatively constant, whereas noise is modeled as a zero-mean white Gaussian. Despite ignoring the scale factor, the EKF filter opts not to utilize these direct measurements during the prediction phase. Instead, it relies on the delta angles (10) and the delta velocities (11), calculated independently for each axis:

(10) $$\Delta {ang}_{x,y,z}=\int_{t}^{t+T_{s}}\omega_{x,y,z}dt,$$

(11) $$\Delta v_{x,y,z}=\int_{t}^{t+T_{s}}a_{x,y,z}dt.$$

Finally, two measurement vectors are formed: $\Delta_{ang}={\left[\Delta {ang}_{x},\Delta {ang}_{y},\Delta {ang}_{z}\right]}^{T}$, $\Delta v={\left[\Delta v_{x},\Delta v_{y},\Delta v_{z}\right]}^{T}$. The state vector x contains biases for both the gyroscope $b_{\omega }$ and the accelerometer $b_{a}$ to allow corrections of delta angles and delta velocities in dynamic state equations.

Quaternion q is essential for the construction of rotation matrix $R_{N}^{B}$ that is required to establish the dynamic model of the UAV motion:

(12) $$\begin{array}{ccc} \dot{q} & = & \frac{1}{2}q⊗\left[\begin{array}{c} 0\\ \Delta_{ang}-b_{\omega }dt \end{array}\right], \end{array}$$

(13) $$\begin{array}{ccc} {\dot{v}}_{N} & = & R_{N}^{B}\left(\Delta v-b_{a}dt\right)-{\left[0,0,g\right]}^{T}dt, \end{array}$$

(14) $$\begin{array}{ccc} {\dot{p}}_{N} & = & v_{N}dt, \end{array}$$

where symbol ⊗ denotes quaternion multiplication.

**Figure 3:**
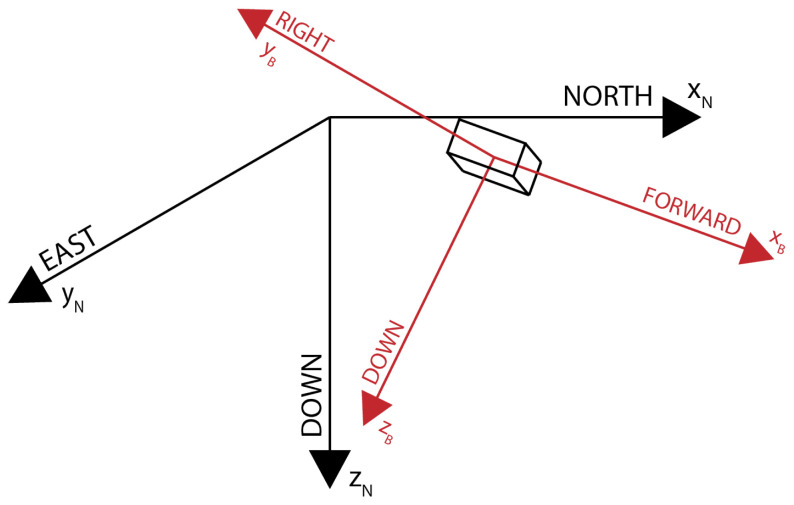
Navigation (black) and Body (red) Frame.

The ECL library uses the local tangent plane coordinates North-East-Down (NED) as the navigation frame and Front-Right-Down (FRD) as the body frame, as shown in Figure 3. Different approaches were chosen to define the navigation and body frames in the rest of the system. Therefore, conversion from the used East-North-Up (ENU) local navigation frame and the Front-Left-Up (FLU) body frame must be performed before injecting sensor data into the EKF filter.

The ECL library is commonly used in many autopilot software projects, such as PX4 Autopilot [36] and ArduPilot [37].

### 2.3. Profiling of a Real-Time Operating System

**Figure 4:**
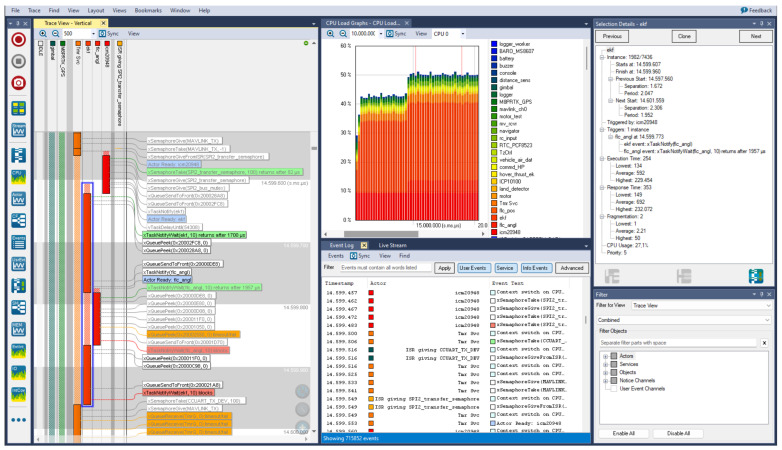
Percepio Tracealyzer profiling example [38].

The UAV firmware is based on the FreeRTOS real-time operating system (RTOS). In every RTOS, the adjustment of priorities and the management of the RTOS functionality for every task running on the microcontroller must be precise. With the help of the Percepio Tracealyzer [38] software in version 4.7.0 used to profile the RTOS runtime, any anomalies can be detected. An example of a recorded trace is shown in Figure 4. In the case of this article, the most important graph for runtime analysis is “Trace View” which is shown on the left of that figure and presented in the form of a Gantt chart. This graph presents the state of every task in the system at a given time and includes specific RTOS events marked with boxes with a description on the right side.

During normal operation, tasks can be in four different states:

- Ready—task is ready to be given CPU time,
- Running—task currently running on CPU, it can be preempt by higher priority task and put back to “Ready” state,
- Blocked—task waiting for blocking event to end,
- Suspended—task manually suspended.

In the Gantt graph, every column represents a single task or interrupt service routine (ISR). For ease of reading the graph, every column has a different color. The “Ready” state of the given task is marked with a transparent pattern, while the solid blocks show the “Running” state of the task. The system scheduler manages and assigns the CPU time to every task by its state and priority.

FreeRTOS includes implementations to track key events, such as context switches between tasks, semaphore lock and unlock, or queue actions. In addition, any interrupt service routines (ISR) can be registered to be monitored, or the user can create other custom events. The recorded trace of events can be used to visualize the working RTOS and bring up valuable data that can speed up the debugging process, optimize performance, and verify timings in embedded software. Tracealyzer supports multiple streaming methods, such as serial port (USB/RS232), TCP/UDP, “to file”, and more.

### 2.4. Experiment Methodology

Our own UAV designs were used in the experiments. These are classic quadrocopters with two different frame sizes, i.e., 13 and 20 inches in diameter. Our design uses 3-blade propellers with a diameter of 7 and 8 inches. The whole thing is powered by 3S batteries. The flight controller, the so-called autopilot, is also a proprietary design similar to PixHawk FMUv5, based on the STM32F7 microcontroller. The autopilot is equipped with a 9-DOF IMU sensor, a barometer, a GNSS receiver, and a ground distance measurement sensor (VL5xx or Terarenger). In addition, the autopilot cooperates with the Raspberry Pi computer onboard, which supports the camera. The most important difference from the PixHawk FMUv5 solution concerns the software, as we use the FreeRTOS operating system. The ECL [27] library (branch “master”, commit “b3fed06”), which implements the EKF filter, has been adapted to our project without changes. This approach makes it easier for us to debug and analyze the processes taking place within the ECL library. In particular, together with Percepio Tracealyzer v4.7.0 software, we gain the ability to precisely control the running times of individual threads of our own prototype autopilot software.

The UAV system allows the recording of all selected data structures, with settable frequency, on the SD card in the form of a logfile that can be replayed and analyzed later in the MATLAB R2023a software. Due to limitations of the SD card bandwidth, only internal data structures or RTOS trace could be saved during flight. As a result of this, the experiments were divided into two phases.

The first phase included tests on the bench with the RTOS trace streamed via USB cable and the data structures saved to an SD card. During the test, the motors were running without propellers and the movement of the drone was simulated by hand. This phase allowed for the identification of issues with the RTOS and the corresponding behaviors in the logged data.

The second phase consisted of flights on multiple UAV platforms with data structures being logged to an SD card. Later, offline analysis was performed, taking into account discovered dependencies from the previous phase. The results are provided in the next section.

## 3. Results

The results of the study can be divided into two groups. RTOS analysis is presented in Section 3.1 and its effect on UAV stabilization is shown in Section 3.2.

### 3.1. Temporal Disturbances

In real-time systems, it is crucial to maintain the timing regime and ensure that all critical threads meet their deadlines. The most important of these are the tasks of acquiring measurements from the IMU sensor, the state estimation thread using the EKF algorithm, and the stabilization task being part of the flight controller. The flight controller relies on periodicity and expects to receive attitude data every 2 ms. Due to disturbances in the computation time, these data may appear up to 10 ms later or, in critical cases, even 70 ms after the deadline.

**Figure 5:**
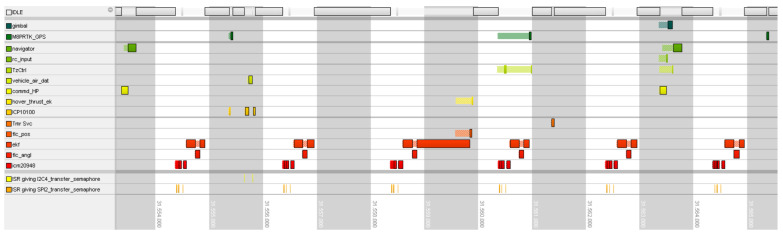
Gantt graph of desired behavior of EKF and rest of the system.

Figure 5 presents the Gantt graph with a view of the RTOS system with only active tasks in a given period of time. The solid blocks in every line show the time of execution of a given task on the CPU core. The transparent pattern appearing before or between solid blocks indicates the thread’s readiness for execution and waiting for the higher-priority task to end. Its appearance between execution blocks signifies the preemption of the thread by a higher-priority process. It is normal to assign lower priority to noncritical tasks to ensure the expected execution timings for critical ones. The following tasks should be considered as most critical in this graph ordered from highest to lowest priority:

- “icm20948”—task for collecting and converting the reading from the IMU sensor,
- “flc_angl”—FC angle stabilization controller process,
- “ekf”—task running the PX4-ECL version of EKF,
- “flc_pos”—FC position stabilization controller process.

The standard cooperation of the four tasks can be divided into the following general steps:

1. reading of data from the IMU sensor (preparation for data transfer, transfer, conversion of received data, sending to other tasks),
2. EKF receives IMU data and performs the prediction step,
3. EKF sends attitude data to FC,
4. FC receives data and creates adequate control outputs for motors,
5. EKF performs the correction step, if any new measurements from other sensors are available for fusion,
6. if EKF is ready, it publishes position data for FC position control,
7. FC receives position data and creates adequate controls.

As angle stabilization is most crucial in UAV flight, the “flc_angl” task can preempt the “ekf” process when the current state of the angles is calculated by it and published for use by the rest of the system. This behavior can be clearly seen in Figure 5, and there are also no unwanted delays in the system.

**Figure 6:**
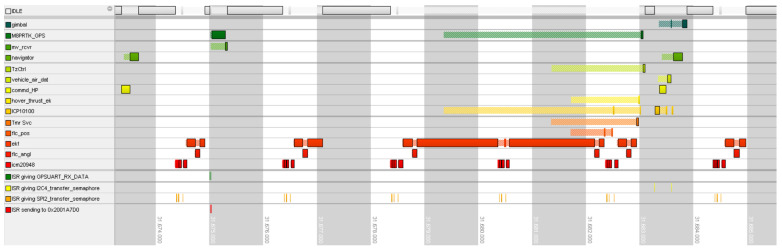
Gantt graph of computational delay of EKF.

Figure 6 shows a problematic scenario where the calculation of the “ekf” task is so lengthy that the rest of the system is holding execution. Only the IMU task with higher priority is able to preempt it to acquire fresh data from sensors, but they are not processed until almost the next reading.

**Figure 7:**
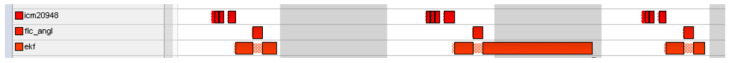
Gantt graph of desired behavior of EKF.

Figure 7 presents two different types of update routines of the EKF filter. The first, the short one, injects IMU data and only makes a prediction step. It takes just under 0.5 ms with IMU data reception and FC calculations. A longer routine also includes a correction step which is performed with a frequency of 100 Hz, and it takes about 1.5 ms.

**Figure 8:**
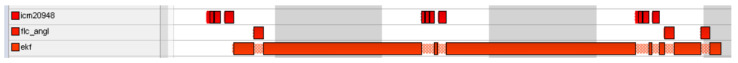
Gantt graph of delay in computation in EKF.

In Figure 8, the execution of the tasks is delayed. There are three samples read, while the second sample is delayed over 2 ms relative to the measurement. Despite the time delay, all samples are included in the EKF calculation and processed.

**Figure 9:**
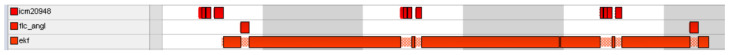
Gantt graph of delay in computation in EKF and lost IMU sample.

The worst recorded case is shown in Figure 9 where, again, the system receives three samples from sensors, but the middle is lost due to the intense computation of the EKF, and also the last one is delayed by about 1 ms.

**Figure 10:**
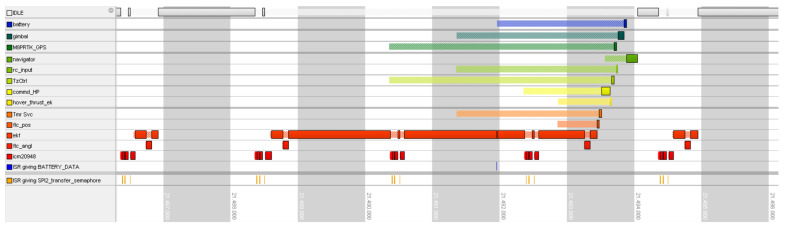
System view of Gantt graph of delay in computation in EKF, lost IMU sample, and influence on the entire system.

As these three tasks are crucial for the functioning and safety of the UAV, they are of the highest priority throughout the system. The situation where the EKF task takes all processor time is reflected in the rest of the system as shown in Figure 10.

The changing of priorities does not solve the problem because the EKF thread still needs to complete its task before other threads can utilize the estimation results.

### 3.2. Effect of Temporal Disturbances on Flight of UAV

The identification of delays in the system resulted in adding to the logging values the time difference “*dt*” between the publications of the estimation results by the EKF algorithm. This allowed for later offline analysis to identify delays in the system during flight.

**Figure 11:**
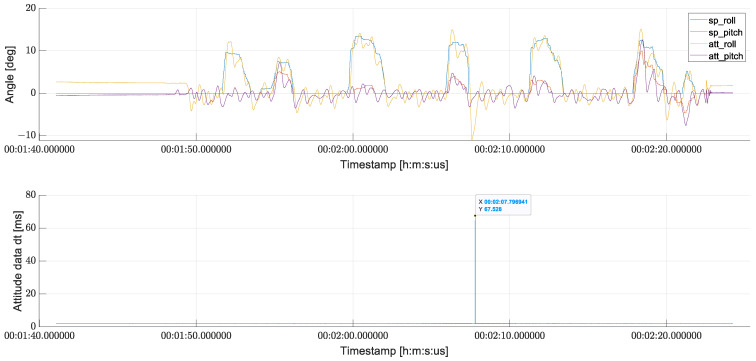
Comparison of measured roll and pitch angles and setpoints from operator (**top**) plotted against time difference between consecutive EKF estimates (**bottom**).

The estimated angles for roll and pitch (marked as “*att_roll*” and “*att_pitch*”) in degrees are presented in Figure 11, as well as the setpoints given by the operator during the flight—“*sp_roll*” and “*sp_pitch*” for roll and pitch angles, respectively. The lower plot shows the difference in milliseconds between the EKF attitude estimations. As can be observed, during flight, all estimation was calculated about every 2 ms with one exception when it took as much as 68 ms to create new data for FC. During this event, the measured error between the given setpoint and the measured angle of the UAV was greater than 10 degrees.

**Figure 12:**
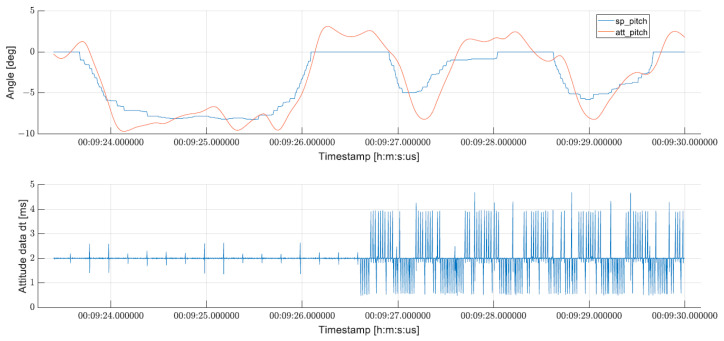
Plot of measured roll and pitch angles, setpoints from operator (top) and time difference between consecutive EKF estimates (bottom).

The measured pitch angles in degrees with the setpoints of the drone operator during manual flight are presented in Figure 12. These results were obtained during a different flight from the one presented in Figure 11. As can be seen in the lower plot, the time differences between consecutive EKF estimates are smaller than 5 ms. Two distinct periods can be identified on the graph—one where the estimation time of successive angles falls within the assumed 2 ms timeframe, and another starting from about 00:09:26.500 when delays in the computations of the EKF algorithm begin to manifest. Subsequent to the onset of these delays, the response to the pitch setpoint noticeably prolongs, accompanied by a substantial increase in error between the desired and measured angles.

Implications of delays smaller than 10 ms are sometimes hard to observe directly during offline analysis but can be “felt” by the operator during manual flight. The UAV seems to be reacting sluggishly to the operator’s input when they occur.

## 4. Discussion

There are challenges in connecting various sensors and tools for problem identification during flight, which can be easily accomplished on the ground. To address this limitation, the authors propose a method of appending “*dt*” to selected data structures to facilitate the identification of temporal disturbances in later offline analysis. Although acknowledging the potential to include more data in the logs, the authors recognize the constraint posed by the limited write speed to the SD card and increased CPU usage while logging data at a higher rate, which is necessary for later analysis.

Disturbances occur irregularly and relatively infrequently, further complicated by the difficulty of replicating them under laboratory conditions.

**Figure 13:**
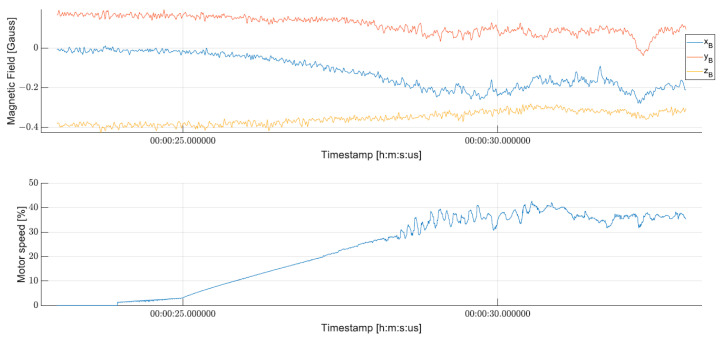
Plot of raw magnetic measurement dependency on motor speed.

Analysis of data obtained from both research and test flights leads to the hypothesis that disturbances in the magnetic field measured by the magnetometer may affect the extended calculations of the EKF algorithm. Motors were observed to generate exceptionally strong interference fields, which vary with motor speed, as depicted in Figure 13, where magnetic field measurements were performed using a magnetometer installed on the autopilot board. The registered magnetic field changes while the motors speed up, despite UAV not moving. The take-off can be observed at around 00:00:28:400, where the motor speed starts to vary to accomplish stabilization. Prior to this point, the registered magnetic field changes by a significant amount. Similar effects may be exerted by external infrastructural objects such as transformers and high-voltage networks, among others. Furthermore, the conducted studies indicate that underground networks also generate magnetic field disturbances.

The ECL library estimates the magnetic field on the UAV, which theoretically should make the EKF resistant to various changes in the magnetic field caused by the above-mentioned noise. The magnetic field of the UAV is represented by the vector $m_{B}$. In the authors’ opinion, the procedure for estimating this field is a potential source of randomly occurring delays. A careful analysis of the data flow diagram of the EKF filter implementation—see Figure 2—indicates that the data from the inertial sensors are downsampled to a frequency of 83 Hz. Then, after new data appear in the FIFO buffer, a prediction step is performed to calculate the position and velocity of the UAV and predict the covariance matrix. Then, the second key step in the EKF algorithm comes, i.e., the step of fusion of data from additional sensors, including the magnetometer. This implementation means that every 10 ms, the average calculation time of the first and second steps of EKF filtration takes over 1 ms instead of 300 μs. This happens due to the accumulation of calculations related to the measurement data fusion block. This is shown very well in Figure 7. However, if the magnetometer measurements are inconsistent compared to current state—taking into account both $m_{E}$ and $m_{B}$—the data fusion step takes much longer time because the ECL library calculates a new value of $m_{B}$. A solution to this issue is the movement of this calculation to another task with lower priority than the EKF thread. Such thread would calculate a new vector $m_{B}$ in the free time, consequently not blocking the main loop of the EKF thread. The drawback of this method is the problematic access to concurrent data which is necessary for both tasks.

The authors propose the mechanisms that allow for continuous monitoring of the behavior of the RTOS system. The problems that prompted the research are significant and lead to emergency flight interruptions. Since the commencement of the research, such significant issues have not occurred thus far. Therefore, it is important to continuously monitor the aforementioned computational disturbances, as their randomness prevents a complete definition of their causes and solutions.

## 5. Conclusions

The research was conducted to identify possible causes of degradation of flight stabilization quality. During experiments, two potential causes for delays were identified and investigated. According to the results of the experiments, the magnetic field disturbances created by UAV motors and external magnetic fields (e.g., power lines) interfere with magnetometer readings and consequently lead to extended EKF time of calculations. The standard practice of shifting the magnetometer as far as possible from the engine does not solve the problem completely. The second identified cause, i.e., serial implementation of the calculation in the PX4-ECL library, which combines magnetometer data with other sensor measurements, can be solved only by changing implementation. Further work will be undertaken in the direction of separating time-consuming calculations of the external magnetic field into a low-priority thread. But such changes are not so simple, because there exists a strict correlation between the current state estimated by the EKF and the external magnetic field which is one of the internal states of the EKF.

## Data Availability

The flight data logs taken during our experiments are published in open database, which collect many similar flight data logs provided by many scientific groups and hobbyists. The address of the database is as follows: https://review.px4.io/browse. Particular logs published in the paper can be found at address: https://review.px4.io/plot_app?log=6b516163-53f1-4887-8886-1217e6cce24a, https://review.px4.io/plot_app?log=45b3c5b9-a569-4468-a6f2-4e25f72031e3, https://review.px4.io/plot_app?log=74632838-320e-4930-918e-3d2e21688e70.
